# Realization of Minimum and Maximum Gate Function in Ta_2_O_5_-based Memristive Devices

**DOI:** 10.1038/srep23967

**Published:** 2016-04-05

**Authors:** Thomas Breuer, Lutz Nielen, Bernd Roesgen, Rainer Waser, Vikas Rana, Eike Linn

**Affiliations:** 1 Peter Grünberg Institut 7, Forschungszentrum Jülich GmbH, 52425 Jülich, Germany; 2 Institut für Werkstoffe der Elektrotechnik II, RWTH Aachen University, 52074 Aachen, Germany; 3 JARA – Fundamentals for Future Information Technology, Jülich, Germany

## Abstract

Redox-based resistive switching devices (ReRAM) are considered key enablers for future non-volatile memory and logic applications. Functionally enhanced ReRAM devices could enable new hardware concepts, e.g. logic-in-memory or neuromorphic applications. In this work, we demonstrate the implementation of ReRAM-based fuzzy logic gates using Ta_2_O_5_ devices to enable analogous Minimum and Maximum operations. The realized gates consist of two anti-serially connected ReRAM cells offering two inputs and one output. The cells offer an endurance up to 10^6^ cycles. By means of exemplary input signals, each gate functionality is verified and signal constraints are highlighted. This realization could improve the efficiency of analogous processing tasks such as sorting networks in the future.

Resistive switching memories (ReRAM) are considered as highly attractive emerging technology to implement future high-density non-volatile memory or storage^1^,^2^,^3^,^4^. ReRAM devices offer low operating voltages, excellent scaling properties and compatibility to 4F^2^ nanocrossbar arrays^5^. In such nanocrossbar arrays either a bipolar selector in series (1S1R)^6^ or a complementary resistive switch (1CRS)^7^ is used to prevent undesired sneak paths.

Besides the memory operation, several logic approaches based on ReRAM devices are suggested to overcome the von Neumann bottleneck^3^,^4^,^8^,^9^. For example, ReRAM cells can be used to implement look-up-tables, replacing SRAM memory^10^,^11^,^12^. Alternatively, ReRAM cell arrays can be used as programmable interconnects, as for example in the famous CMOL/FPNI FPGA concept^13^,^14^. Structures consisting of two ReRAM devices are used in various FPGA-like concepts either in serial or anti-serial configuration^15^. A two-input-one-output ReRAM structure is used for routing^16^, structural identical to the complementary resistive switch structure with accessible middle electrode which we use for implementation of MIN/MAX gates (Fig. 1). The approach which we follow in this paper uses ReRAM devices directly as logic operating device. Boolean logic approaches falling in this category are the (material) implication logic^17^ and CRS logic^8^,^18^,^19^, for example. Both approaches are in principle compatible to crossbar arrays when either 1S1R or 1CRS is used^20^. Beyond that, the CRS logic concept^8^,^19^ features a computing-in-memory approach to overcome the von-Neumann bottleneck^21^,^22^. From a theoretical perspective, Šuch and Klimo suggested to use two-memristor-circuits to implement Minimum and Maximum gates^23^. In general, one of these three-terminal devices has two inputs and detects the higher or lower input voltage. For binary considerations these devices are equivalent to logical AND/OR gates^24^. These gates could be used in analogue signal processing and could help to realize small-size sorting networks^25^ taking some limiting properties into account^26^. The proposed gate structure consists of two anti-serially connected devices, i.e. the device stack is very similar to a conventional complementary resistive switch, but offers a third terminal at the middle electrode^16^,^27^,^28^.

Recently, we have demonstrated and characterized such three terminal CRS devices^29^. Šuch *et al.* have shown that the behavior of real memristive devices strongly diverges from ideal memristor behavior. However, a proper Minimum or Maximum gate functionality is enabled by adding some additional constraints in terms of input signal amplitude^28^. Nevertheless, being aware of those constraints, which will be discussed in detail, ReRAMs are well suited for the implementation of memristive fuzzy logic gates. The feasibility of both Minimum and Maximum function is experimentally demonstrated in this paper by using integrated CRS devices, which offer an access to the middle electrode.

## Concepts

### CRS mechanism and the logic function

**Figure 1:**
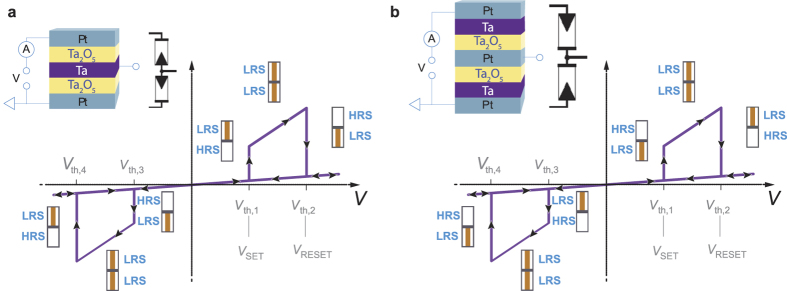
(**a**) CRS stack, equivalent circuit and switching scheme for Minimum gate. (**b**) Is the reversed CRS stack with corresponding equivalent circuit and the resulting switching scheme for Maximum gate. Next to the purple colored ideal *I*-*V* curve, the actual CRS state is indicated by a resistance scheme. The threshold voltages *V*_th,1_, *V*_th,2_, *V*_th,3_ and *V*_th,4_ specify where the BC/TC resets/sets.

The CRS device is based on two anti-serial integrated ReRAM cells, called bottom cell (BC) and top cell (TC). Each cell can toggle between a high resistive state (HRS) and a low resistive state (LRS). Here, two Ta_2_O_5_-based ReRAMs are used as illustrated in Fig. 1. The active switching Ta_2_O_5_ layer of each cell is sandwiched between a Pt and a Ta electrode. The final CRS stack is symmetric. Therefore, the polarity of the *I*-*V* characteristic does not depend on which electrode (top Pt or bottom Pt) the voltage is applied. Due to the anti-serial stacking the two single cells always switch complementarily. Figure 1 depicts the two possibilities of vertical stacked CRS devices. The resulting *I*-*V* curves for both cases are indistinguishable. However, the voltage polarity, where BC and TC resets or sets, differs for both stacks. Figure 1a shows the more common CRS layer stack (Pt/Ta_2_O_5_/Ta/Ta_2_O_5_/Pt). Starting with the CRS state LRS/HRS (TC in LRS and BC in HRS), a positive voltage is applied to the top Pt, whereas the bottom Pt is grounded. At threshold voltage *V*_th,1_ the BC sets to LRS and the whole device switches to the transition state LRS/LRS. Increasing the voltage to *V* > *V*_th,2_ the TC resets, i.e. the CRS switches to HRS/LRS. If a positive voltage is applied again, the CRS state will not change anymore and stays in HRS/LRS. Only by applying a negative voltage, the CRS devices switches to the transition state LRS/LRS, since the TC sets at *V*_th,3_. Finally, at *V*_th,4_, the devices switches back to the initial state LRS/HRS by resetting the BC. The second CRS stack in Fig. 1b offers a reversed switching of BC and TC in comparison to Fig. 1a, but this is not observed in the *I*-*V* characteristic as both stacks offer the same behavior.

Only the two non-transition CRS states (LRS/HRS, HRS/LRS) are interesting for the logic functions. The crucial points for the implementation of Minimum (MIN) and Maximum (MAX) logic are:

- Dependent on the applied voltage, the stacked device offers reversible toggling between two resistive states (which is offered by the CRS).
- In both CRS states, one cell is always in the high resistance state, whereas the other one is in the low resistive state.
- The resistance of one cell being in the HRS is always much higher than the resistance of the other cell being in the LRS.

**Figure 2:**
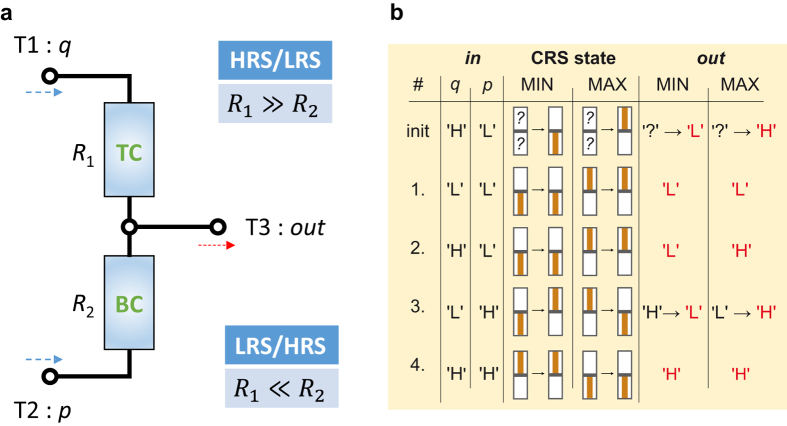
(**a**) Generalized measurement schema for the MIN/MAX function. (**b**) Truth table: MIN/MAX function for the different conditions of input signals *q* and *p* (2^nd^ and 3^rd^ column). The signals *q* and *p* are applied to the respective terminals T1 and T2. That is either a low potential (‘L’) or a high potential (‘H’). At T3 the output voltage *out* (either ‘L’ or ‘H’) is detected, which depends on the gate function and the inputs for *q* and *p*. In general, an initialization step is not required. However, this step is here performed to predict the observed switching behavior of the CRS device (4^th^ and 5^th^ column). The actual switching process depends on the previous state, the final result (6^th^ and 7^th^ column, red labeled) is independent though.

For the MIN/MAX logic, the CRS is here considered as a three-terminal (T1, T2 and T3) gate device as illustrated in Fig. 2. Two terminals (top and bottom Pt) are used for the input signals *q* and *p*. The third terminal (middle electrode) is used for the output signal. During the application, T1 and T2 is either set to low potential ‘L’ or to high potential ‘H’. At first, two static resistors instead of TC and BC with *R*_1_ and *R*_2_ are assumed, to understand more easily, how the device behaves in sense of the gate logic. The voltage *out* detected at T3 is given by


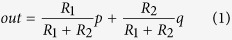


For *R*_1_ ≫ *R*_2_ Equation (1) is simplified to *out ≈ p*. This can physically be interpreted as follows. The voltage applied to the T1 drops completely across *R*_1_ and the output voltage measured at T3 is equivalent to the input voltage at T2 (independently of high or low potential). Therefore, the MIN/MAX gate functionality cannot be implemented in this static configuration (by classical resistors). However, the CRS device offers two anti-serially integrated dynamic resistors. Depending on the current CRS state and the applied signals to T1 and T2, it switches from LRS/HRS to HRS/LRS and vice versa. This switching property allows the implementation of the MIN/MAX operation and is described in detail in the following sections.

Figure 2b depicts the truth table for the MIN/MAX functions. It illustrates the device dynamics for all possible combinations of the applied *in* signals *q* and *p* and the resulting *out* signal. First, the CRS is initialized by setting *q* to ‘H’ and *p* to ‘L’, which is equivalent to applying a positive voltage to the top Pt, whereas the bottom Pt is grounded (cf. Fig. 1). This step is only performed at the beginning and not repeated for each combination of *q* and *p*. Consider, the initialization is not required for the demonstrated logic application, but it facilitates the verification and the gate dynamics in an easier way. Secondly, the observation of the switching event depends on the previous state (within the time resolution of the used measurement equipment). Nevertheless, the final device state and the linked *out* signal is specific for each combination of *q* and *p*. The MIN function is realized by the CRS stack illustrated in Fig. 1a. Initialization toggles the stack into the HRS/LRS state. In the first step, *q* = ‘L’ and *p* = ‘L’ are applied to T1 and T2. In total, no voltage drops across the complete stack, therefore TC remains in the HRS, whereas BC is in the LRS. The signal applied to T1 drops completely across the TC (being in HRS) and the output at T3 is equivalent to the input voltage applied to T2. Next, two asymmetric signals *q* = ‘H’ and *p* = ‘L’ are applied. This is identical to the initialization step and the CRS device does not switch, therefore *out* = *p* = ‘L’ is measured at T3. In the third step, the polarities at T1 and T2 are switched to *q* = ‘L’, *p* = ‘H’. This is equal to the condition, where in Fig. 1a, a negative voltage is applied to T1, whereas T2 is grounded. The TC toggles from the HRS to the LRS and the BC makes transition from the LRS to the HRS (→ LRS/HRS). After switching of the CRS state, the applied voltage at T2 drops completely across the BC (being in HRS). Therefore, the output signal at T3 is equal to the voltage applied at T1 (*q* = ‘L’), since there is hardly any voltage drop across the TC (being in LRS). For the condition *q* = ‘H’ and *p* = ‘H’, there is again no voltage drop across the whole device stack. Therefore, the CRS stays in the LRS/HRS state and *out* = *q* = ‘H’ is measured at T3. For each combination of *q* and *p,* the MIN gate device always delivers the minimal applied potential as the output signal.

Figure 1b shows the CRS stack, which is used for the implementation of the MAX gate function. The initialization, applying *q* = ‘H’ and *p* = ‘L’ switches the device to the LRS/HRS state. This is due to the reversed stacking of the two cells. For the trivial condition *q* = ‘L’ and *p* = ‘L’, *out* = *q* = ‘L’ is measured, since no switching is expected. Applying *q* = ‘H’ and *p* = ‘L’ does not change the device state and at T3, the signal *out* = *q* = ‘H’ is detected. In the third step with *q* = ‘L’ and *p* = ‘H’ the CRS toggles to the HRS/LRS state so that *out* = *p* = ‘H’ is measured at T3. For the last condition, where *q* = ‘H’ and *p* = ‘H’ is applied to T1 and T2, there is not any voltage drop across the CRS stack, which remains in the HRS/LRS state. Hence, ‘H’ measured at the output terminal T3.

## Results

### Device Characterization

**Figure 3:**
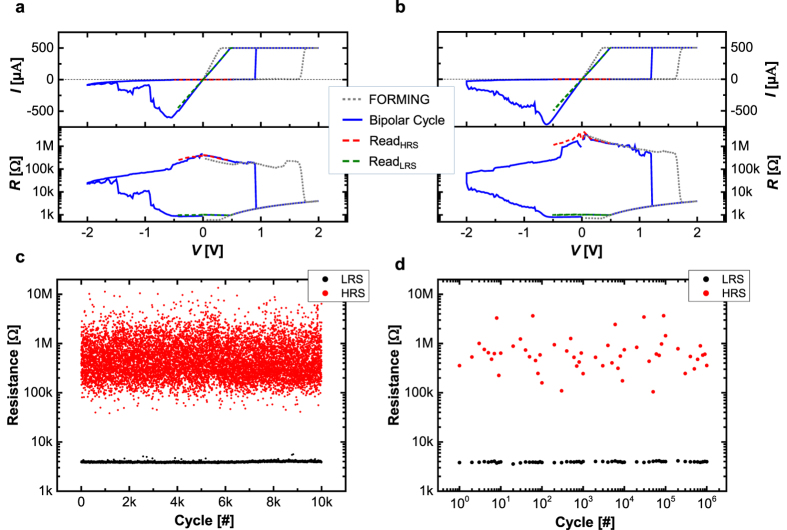
Electrometric, quasi-static characterization of single TC (**a**) and BC (**b**). The upper graph in (**a,b**) shows the *I-V* curve, whereas the lower one shows the derived *R-V* characteristic. Prior to any bipolar switching cycle (blue line), the TC and BC are formed separately (grey dotted line). Additionally, reading sweep after RESET (red dashed line) and reading sweep after SET (green dashed line) are shown. (**c**,**d**) show the endurance measurement data on single bipolar switching cell. Complete endurance data points up to 10^4^ cycles (**c**) and logarithmically measured endurance data points up to 10^6^ cycles (**d**).

The three-terminal CRS device offers an access to the middle electrode (T3). Applying the voltage to T3 and grounding T1 allows to perform separate electroforming and bipolar switching on the TC only. The same applies for the BC by using the electrodes T3 and T2. Figure 3 summarizes the electrometric characterization of the TC and the BC. Figure 3a corresponds to the TC and Fig. 3b is related to the BC. The graphics are split up into the top graph showing the *I*-*V* curve on the linear scale and the bottom graph demonstrating the *R*-*V* curve on the logarithmic scale. The grey dotted line indicates the electroforming on the single cell. The initial resistance *R*_ini_ is in the range of several hundred kΩ and few MΩ. Around 1.7 V, the current increases abruptly due to the formed conductive filament and is limited by the instrumental current compliance (CC) at 500 μA. The CC limits the conductivity of the cell. Lower CC decreases the maximal operation current in the single bipolar switching cell and in the final CRS device^29^. The cell is formed into the LRS around 1 kΩ and offers an ohmic *I*-*V* and *R*-*V* characteristic. The blue line describes the bipolar switching cycle. The gradual RESET process starts by applying negative voltage less than −0.6 V. Finally, the cells end up in the non-ohmic HRS resistance *R*_HRS_ = ~100 kΩ…~1 MΩ. The abrupt SET occurs for positive voltage polarity around 1 V. The ohmic LRS resistance *R*_LRS_ is around 1 kΩ. After the RESET and SET process respectively, the read operation is performed by sweeping the voltage to ±0.5 V (red/green dashed line). The read data shows a similar (positive/negative polarity) resistance ratio of *R*_OFF_/*R*_ON_ ≈ 100…1000, which is sufficient for the MIN and MAX logic operation regarding the considerations in the previous section. Additionally, an endurance test has been performed by microsecond pulses (c, d). The cell resistance has been measured after RESET and SET process respectively by applying a read voltage *V*_read_ = 0.2 V. Figure 3c depicts the complete data points up to 10^4^ cycles exhibiting a clear window without using any target resistance checking algorithm. Figure 3d shows the endurance test up to 10^6^ cycles with logarithmically measured points.

**Figure 4:**
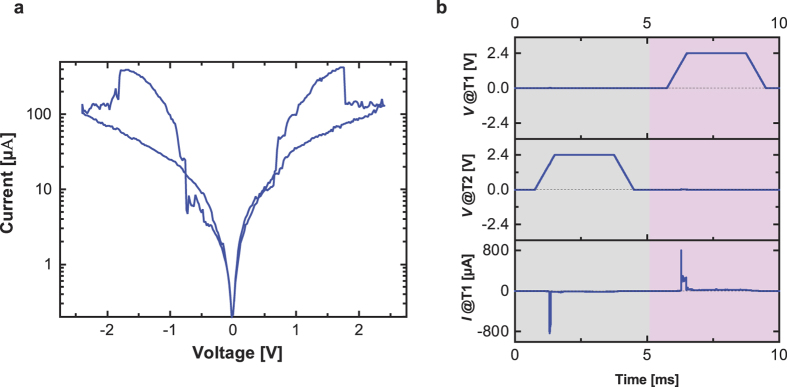
Electrometric characterization on the complete CRS stack. (**a**) Quasi-static *I-V* sweep and (**b)** transient current measurement with millisecond voltage pulses.

To achieve the self-limiting and non-linear *I*-*V* illustrated in Fig. 1 the CRS contact mode is needed, i.e. the forcing voltages are applied to T1 and T2. Figure 4a depicts the quasi-static *I-V* sweep on the total CRS stack, where the voltage is applied to T1, whereas T2 is grounded. The *I*-*V* curves are similar for both gate devices. Due to the controlled separate forming procedure by using low CC, the maximal operation current is less than 500 μA. In contrast to the bipolar switching, the CRS operation inherently offers the current-self-limiting function. The symmetric *I*-*V* clearly shows SET and RESET events of the BC and TC for positive and negative voltage polarity as described in the previous section. Figure 4b shows the transient currents at pulse amplitude of 2.4 V and width of few milliseconds. The pulse behavior is more significant for the real applications than the quasi-static performance. During the pulse characterization, the CRS state switches by the voltage stimulus and the response is detected as the current peak. These Ta_2_O_5_-based CRS devices offer high endurance up to 10^6^ switching cycles^29^.

### MIN Gate Function

**Figure 5:**
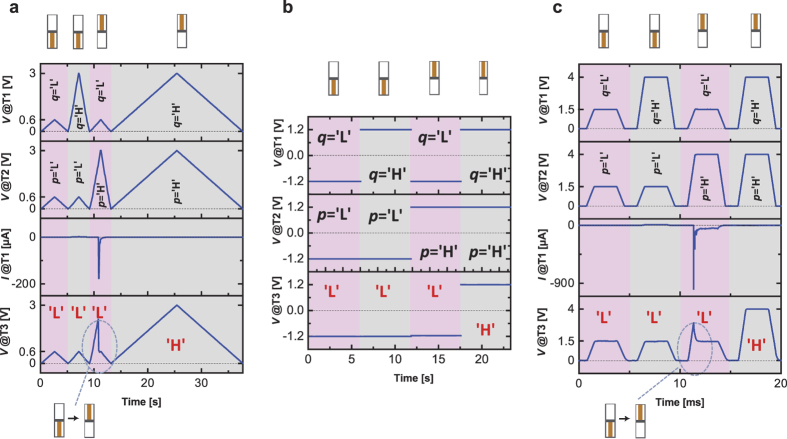
The MIN operation is implemented by three different voltage modes: quasi-static voltage sweep (**a**), base voltage (**b**) and voltage pulse (**c**). The resistance scheme at the top indicates the final CRS state. If a change of the CRS state is observable in the measurement, the switching is illustrated explicitly by the resistance scheme at the bottom. The graphs show from top to bottom: voltage signal lines at T1 and T2, current signal line (only (**a,c**)) and the detected voltage signal at T3. (**b**) does not include the current signal line, since no switching dynamics are detected. ‘H’ (high potential) and ‘L’ (low potential) refer to the applied voltage level and the recorded output signal, respectively. ‘L’ and ‘H’ are not fixed to any certain values as demonstrated by the three examples (**a–c**).

Exemplary implementations of the MIN function are summarized in Fig. 5. Additional measurements are attached in the Supplementary Information. The logic operation is realized by three different voltage modes: voltage sweep (a), base voltage (b) and voltage pulse (c). The experiment is conducted for different voltage values for ‘L’ and ‘H’ to demonstrate the flexibility of the gate terminal. The maximal applied voltage, given by the difference of high and low potential, is the crucial point for toggling the states.

The dynamic and static behaviors of the CRS devices are plotted as a function of time. The resistance scheme at the top indicates the final CRS state. The upper two signal lines show the voltages at T1 and T2. The third line in Fig. 5(a,c) represents the measured current, whereas the lowest signal line shows the *out* voltage at T3. If a change of the CRS state is observable in the measurement (abrupt voltage change or current spike), the switching is highlighted and illustrated explicitly by the resistance scheme at the bottom. Initially, the CRS device is set to the initial state by applying ‘H’ to T1 and ‘L’ to T2. The initialization is not shown, since it is not required for the Minimum function. However, this process allows to verify the correct behavior of *out* at T3 for the first condition of the inputs *q* and *p*. The same sequence of the four different combination of *q* and *p* is used as introduced in the truth table of Fig. 2b. Consider, the demonstrated sequence does not have any impact on the final logic result. However, it could have an impact on the switching dynamics of the *out* signal at T3. The outputs are independent of the voltage mode for a certain combination of *q* and *p*. In each case, the device delivers the minimum value of the two inputs and behaves as the MIN gate. Figure 5b shows the base voltage mode operation of the CRS device (applying of constant voltage levels). There is no current signal shown, since switching dynamics takes place in the rising ramp and are not detected. Nevertheless, for *q* = ‘L’ and *p* = ‘H’ the CRS device changes the states. Due to the abrupt voltage increase of the applied base voltages and the limited time resolution, the switching is not detected by the experimental equipment. The switching dynamic in the measured current (spike) and the voltage at T3 is observable in Fig. 5(a,c). During sweeping *q* to 0.6 V and *p* to 3 V, the CRS device initially does not change the state, shown in Fig. 5a, since the total applied voltage is too low and the voltage at the T3 increases towards 3 V instead of 0.6 V. Whenever the total applied voltage is sufficient, the CRS toggles from HRS/LRS to LRS/HRS and the voltage at T3 drops abruptly to 0.6 V. An equal sudden voltage drop is observed for the pulsed mode (Fig. 5c), as the CRS device changes the state.

The long sweeping time in Fig. 5a for *q* = ‘H’ and *p* = ‘H’ (3 V) in comparison to the other previous cases is only due to the experimental setup. The automatically adjusted sweeping rate depends on the measured current level. For the condition *q* = *p,* no voltage drops across the complete stack and only noise current is detected during the voltage sweeping. Hence, the same is true for the condition *q* = *p* = ‘L’ (0.6 V). Here, the sweeping time is five times shorter for the last case, since the voltage amplitude is also five times lower.

### MAX Gate Function

**Figure 6:**
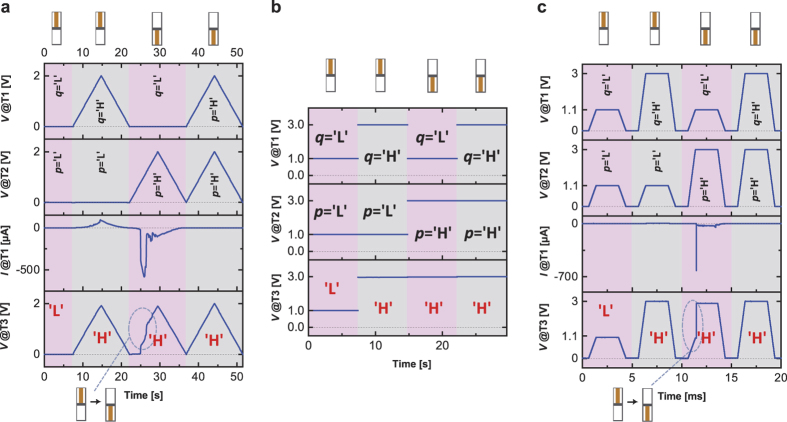
The MAX gate function is implemented by three different voltage modes: quasi-static voltage sweep (**a**), base voltage (**b**) and voltage pulse (**c**). The resistance scheme at the top indicates the final CRS state. If a change of the CRS state is observable in the measurement, the switching is illustrated explicitly by the resistance scheme at the bottom. The graphs show from top to bottom: voltage signal lines at T1 and T2, current signal line (only (**a,c**)) and the detected voltage signal at T3. (**b**) does not include the current signal line, since no switching dynamics are detected. ‘H’ (high potential) and ‘L’ (low potential) refer to the applied voltage level and the recorded output signal, respectively. ‘L’ and ‘H’ are not fixed to any certain values as demonstrated by the three examples (**a–c**).

The MAX gate function is realized by the reversed CRS stack (cf. Fig. 1b). Analogous to the MIN gate, the CRS device is also initialized, although it is not essential for the correct operation. Figure 6 summarizes the results for the voltage sweep (cf. Fig. 6a), the base voltage (cf. Fig. 6b) and the voltage pulse mode (cf. Fig. 6c). To demonstrate that the MAX gate does not work exclusively for fixed input values for ‘L’ and ‘H’, different low and high potentials are applied. The composition of Fig. 6 is completely analogous to the previous MIN function. The *out* signal always delivers the maximal value of *q* and *p*, regardless of the applied voltage input signal. The Supplementary Information includes further examples. The initialization voltage polarity and the sequence for *q* and *p* have been changed to demonstrate that there is not any impact on the final result.

## Discussion

As experimentally shown, the result of the MIN and MAX operation is not directly available at the output *out*, but a certain settling time of the gate is required. Mainly, the settling time depends on the voltage amplitude applied to T1 and T2, which means that for small signals or ramp signals with slow slew rate MIN and MAX operation will take longer than for fast pulses offering larger voltage amplitudes. The demonstrated pulse driven application is limited by the impedance converter, which supports a bandwidth of 8 MHz and a slew rate up to 2.8 V/μs. The MIN/MAX gates have been operated in the millisecond-pulse range, since it guarantees the detection of abrupt voltage changes occurring as a result of the switching dynamics. However, sensing the switching kinetics is not required as long as the CRS device works properly. The switching dynamics have been shown merely to demonstrate the functionality of the device logic. The gate device does not exhibit any limitation regarding the operation speed, since the ReRAM offers a feasible switching (write operation) speed below 200 ps^30^. Therefore, the challenge is to optimize the sensing of the *out* voltage signal. An impedance converter with high input resistance, wide bandwidth and high slew rate would be an optimal sensing device. However, this quality of circuitry is rather uncommon for integrated sense amplification and stages with poorer input impedances are established. So in general, the input impedance of elements of the next logic stage has an impact on the current stage, i.e. the loading of the *out* voltage signal has to be considered for circuit design. For many intended applications like audio signal processing the timing constraint is relaxed since the operation speed of the analogue gates in the range of microsecond would be sufficient.

The value of ‘L’ or ‘H’ is not theoretically limited (consider, the impedance converter supplies a limited *out* voltage; here: ±12 V). However with respect to the considerations in the section about the concepts, a limitation regarding the gate operations is given by the input difference of the low and the high potential. The operation voltage given by this difference has to be at least equal or larger than *V*_th,2_ and *V*_th,4_, respectively. Otherwise the CRS would behave as static and cannot meet all requirements for the MIN/MAX gate function. Furthermore, the potential difference, especially for the pulse mode, has to be considered, since the total voltage drop determines the switching kinetics, which is dominated by the non-linear voltage-time characteristic of the ReRAM^31^,^32^,^33^,^34^. The voltage drop across the device has to be sufficient so that the CRS device switches at the given pulse width. Further optimization of input signal differences could be achieved by modifying other pulse parameters, e.g. rising and falling time. Another approach is to decrease the minimal required operation voltage (*V*_th,2_ and *V*_th,4_) by material engineering of ReRAM devices.

In terms of concatenability, the presented MIN and MAX logic gates offer limited performance since there is no signal restoration within the memristive device. One option is to add analog buffers in the circuitry. However, small circuit blocks without buffers will work properly, for example implementation of area and energy efficient memristive sorting networks. Alternatively, one can use a clocked transistor-based scheme for cascading^16^. This scheme also enables proper forming.

Compared to conventional CMOS approaches, the presented ReRAM approach offers smaller unit array and superior scaling properties. A basic requirement to keep the power consumption low is to use ReRAM devices offering large high resistive states (HRS) and fast switching from LRS/HRS to HRS/LRS. In general, further improvements of ReRAM cell performance in terms of reliability, cycle-to-cycle variance and endurance are required to enable ReRAM based memory and logic applications. Although ReRAM devices enable energy-efficient operations in principle^35^, the question whether the energy-efficiency of ReRAM-type MIN/MAX gate-based circuits is better than comparable CMOS circuit cannot be answered without knowing the area of application (e.g. sorting or audio signal processing) and actual circuit implementation.

## Conclusion

In this work, we have demonstrated the MIN and the MAX gate functionality in Ta_2_O_5_-based memristive devices offering an endurance up to 10^6^ cycles. In contrast to ideal memristors, the input voltage difference is the crucial parameter since settling time of the output signal strongly dependents on the input signal difference. Technologically, the MIN and MAX gates can be directly derived from integrated CRS devices by adding an access wire to the middle electrode. Due to the ultra-small gate size interesting analogous processing tasks such as sorting networks could be implemented efficiently in the future.

## Methods

### Device Fabrication

Two types of devices have been fabricated, one for the MIN gate and the other one for the MAX gate. In both cases, the starting point is a thermally oxidized p-type Silicon wafer. Firstly, 5 nm Titanium (Ti) (as adhesion layer) and 30 nm Platinum (Pt) are deposited by sputtering. Next, these layers are patterned with the bottom electrode layer. This is achieved by covering the sample with photoresist, patterning the resist by photolithography and transferring the resist pattern into the metals by chemical and physical dry etching. For the MIN gate device, 10 nm-thick Tantalum oxide (Ta_2_O_5_) and 10 nm-thick Tantalum (Ta) are deposited. Both layers are patterned as middle electrode. At last, 10 nm-thick Ta_2_O_5_ and 25 nm-thick Pt are deposited and patterned as top electrode. The SEM image of the vertical CRS stack for the MIN gate is shown in Supplementary Fig. S1a. The three in- and output terminals (T1, T2 and T3) are displayed additionally. The number next to the T1 and T2 contact pads indicates the line width.

For the MAX gate device, a planar CRS device has been fabricated by connecting two single cells anti-serially. In contrast to the vertical stack, which is also possible here, planar CRS structure is easier to realize and requires less processing steps. After the bottom electrode layer is patterned (due to the planar CRS structure, this Pt layer serves as the middle electrode contact T3 in the final device; cf. Supplementary Fig. S1b), 10 nm-thick Ta_2_O_5_, 10 nm-thick Ta and 25 nm-thick Pt are deposited. These films are structured by the top electrode layer (Due to the planar CRS structure, one Pt contact is used as top electrode T1 and the other one as bottom electrode T2; cf. Supplementary Fig. S1b). The SEM image of the MAX gate device is shown in Supplementary Fig. S1b.

Deposition of Ta, Ti and Pt is performed by DC sputtering using a corresponding pure metal target and argon as sputtering gas, whereas the Ta_2_O_5_ thin film is grown by RF reactive sputtering using the Ta target and an oxygen-argon gas mixture. The process pressure is always around 2 × 10^−2^ mbar.

### Electrical Characterization

All electrometric measurements (quasi-static and pulsed) have been performed on the Keithley 4200-SCS and the Agilent B1500A. For quasi-static measurements the *out* voltage at T3 is detected by a voltage measuring unit (using the current bias mode). Here, the current level for T3 is fixed at 0 A. During the measurement the system applies an inverse voltage at T3 to keep the current there on the 0 A level. The inverse voltage is equal to the resulting voltage of the signals applied at T1 and T2. However, this method cannot work for pulsed signals, since regulation of the inverse voltage is too slow. Therefore, the voltage at T3 is measured by the self-made impedance converter and monitored by the Keithley 4200-SCS. The impedance converter exhibits a bandwidth of 8 MHz and a slew rate up to 2.8 V/μs. The high input resistance of 3 GΩ supports that almost no current flows through T3.

## Additional Information

**How to cite this article**: Breuer, T. *et al.* Realization of Minimum and Maximum Gate Function in Ta_2_O_5_-based Memristive Devices. *Sci. Rep.* **6**, 23967; doi: 10.1038/srep23967 (2016).

## Supplementary Material

Supplementary Information
