# Supplementary material for: Realization of Minimum and Maximum Gate Function in Ta2O5-based Memristive Devices
Source: Sci Rep. 2016 Apr 5;6:23967. doi: 10.1038/srep23967 (PMC4820708; doi:10.1038/srep23967)
Supplement: Supplementary Information [file srep23967-s1.pdf]

SUPPLEMENTARY INFORMATION

Realization of Minimum and Maximum Gate Function in Ta_2O_5-based Memristive Devices

Thomas Breuer[1,3], Lutz Nielen[2,3], Bernd Roesgen[1,3], Rainer Waser[1,2,3], Vikas Rana[1,3], Eike Linn[2,3]

[1] Peter Grünberg Institut 7, Forschungszentrum Jülich GmbH, 52425 Jülich, Germany

[2] Institut für Werkstoffe der Elektrotechnik II, RWTH Aachen University, 52074 Aachen, Germany

[3] JARA – Fundamentals for Future Information Technology, Jülich, Germany

Corresponding author: Dr. Eike Linn (linn@iwe.rwth-aachen.de)

Supplementary Section 1

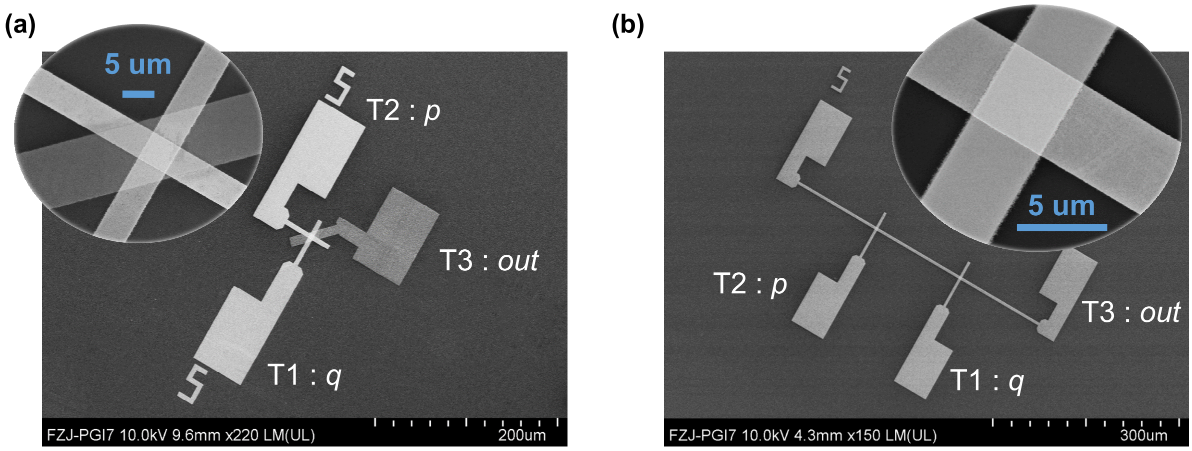

Figure S1 SEM image of the final CRS devices. A vertical stack solution is used for the MIN gate device (a), whereas a planar device concept is applied for the MAX gate device (b). T1, T2 and T3 indicates the contact terminals for the in- and output signals used for the MIN/MAX implementation.

Supplementary Section 2

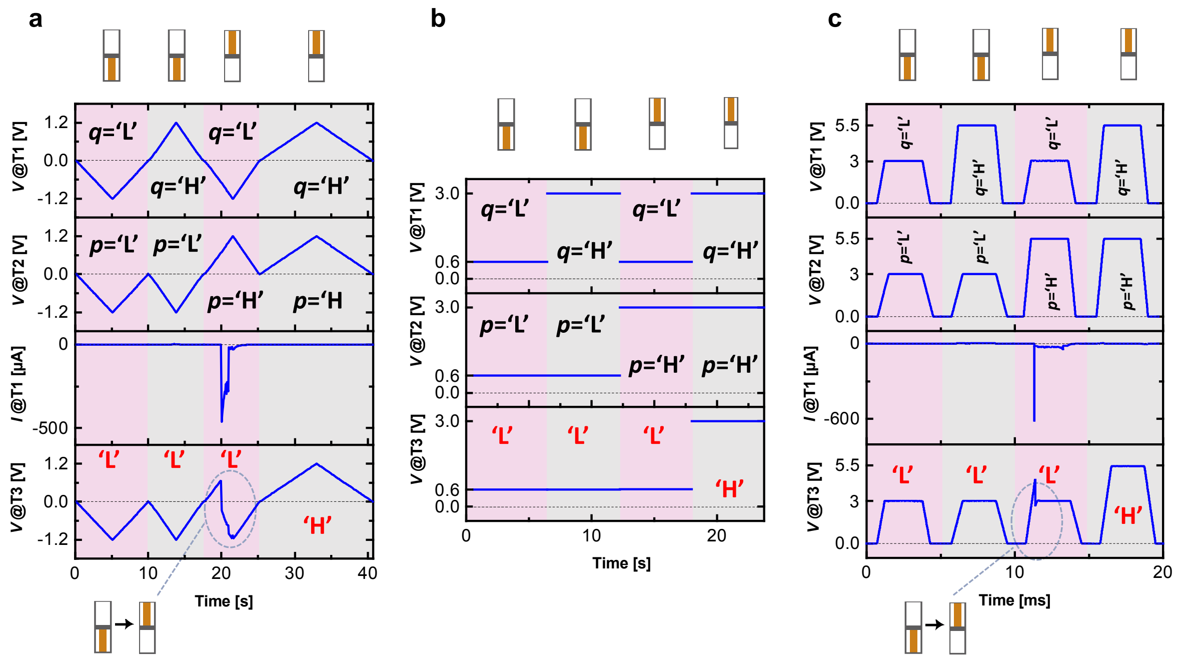

Figure S2 The MIN operation is implemented by three different voltage modes: quasi-static voltage sweep (a), base voltage (b) and voltage pulse (c). The resistance scheme at the top indicates the final CRS state. If a change of the CRS state is observable in the measurement, the switching is illustrated explicitly by the resistance scheme at the bottom. The graphs show from top to bottom: voltage signal lines at T1 and T2, current signal line (only a, c) and the detected voltage signal at T3. (b) does not include the current signal line, since no switching dynamics are detected. ‘H’ (high potential) and ‘L’ (low potential) refer to the applied voltage level and the output signal, respectively.

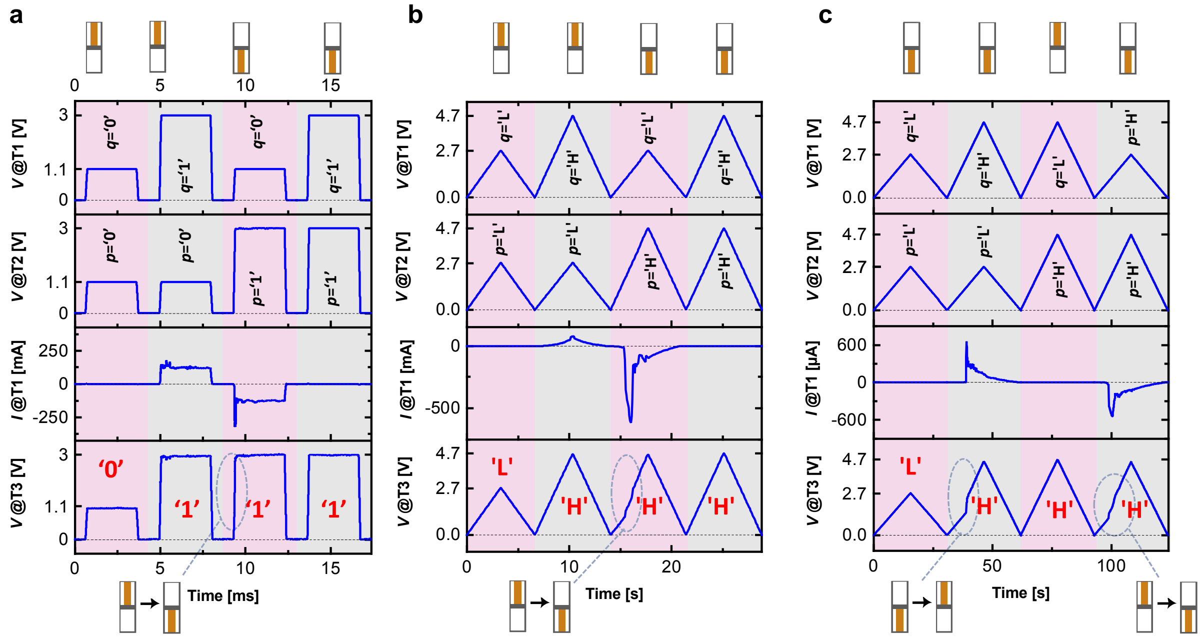

Figure S3 The MAX gate function is implemented by different conditions: voltage pulse (a), quasi-static voltage (b, c). Additionally, (b) and (c) distinguish from each other by the reversed initialization voltage polarity and by a modified sequence for p and q. The resistance scheme at the top indicates the final CRS state. If a change of the CRS state is observable in the measurement, the switching is illustrated explicitly by the resistance scheme at the bottom. The graphs show from top to bottom: voltage signal lines at T1 and T2, current signal line and the detected voltage signal at T3. ‘H’ (high potential) and ‘L’ (low potential) refer to the applied voltage level and the output signal, respectively.
